# Effect of vitamin D on endothelial and ventricular function in chronic heart failure patients: A prospective, randomized, placebo-controlled trial

**DOI:** 10.1097/MD.0000000000029623

**Published:** 2022-07-22

**Authors:** Jong Shin Woo, Yeongmin Woo, Jeong Yoon Jang, Sang Jin Ha

**Affiliations:** a Division of Cardiology, Department of Internal Medicine, Kyung Hee University Hospital, Kyung Hee University, Seoul, Republic of Korea; b Division of Cardiology, Department of Internal Medicine, Gangneung Asan Hospital, University of Ulsan College of Medicine, Gangneung, Republic of Korea; c Division of Cardiology, Department of Internal Medicine, Gyeongsang National University Changwon Hospital, Changwon, Republic of Korea.

**Keywords:** chronic heart failure, vitamin D, endothelial function, endoPAT, ventricular function

## Abstract

Low 25-hydroxyvitamin D (25OHD) levels are common in patients with chronic heart failure (HF) and are associated with increased mortality risk. This study aimed to establish the safety and efficacy of oral vitamin D3 (cholecalciferol) supplementation and its effect on endothelial and ventricular function in patients with stable HF.

**Methods:**

This study was an investigator-initiated, multicenter, prospective, randomized, placebo-controlled trial. Seventy-three HF patients with 25OHD levels < 75 nmol/L (30 ng/mL) were randomized to receive 4000 IU vitamin D daily or a placebo for 6 months. The primary endpoint was a change in endothelial function between the baseline and after 6 months as assessed using EndoPAT. Secondary endpoints included changes in echocardiographic parameters and differences in quality of life (6-min walking test and New York Heart Association functional status) at 6 months.

**Results:**

There were no adverse events in either group during the study period. Vitamin D supplementation did not improve endothelial dysfunction (EndoPAT: baseline, 1.19 ± 0.4 vs 6 months later, 1.22 ± 0.3, *P* = .65). However, patients’ blood pressure, 6-min walking distance, and EQ-5D questionnaire scores improved after vitamin D treatment. In addition, a significant reduction in the left atrial diameter was observed.

**Conclusion:**

A daily vitamin D dose of 4000 IU for chronic HF appears to be safe. This dosage did not improve endothelial function but did improve the 6-min walk distance, symptoms, and left atrial diameter at 6 months.

## 1. Introduction

Chronic heart failure (HF) remains the only cardiovascular disease with an increasing hospitalization burden, resulting in high healthcare expenditure. The pathophysiological mechanism underlying HF is complex, and recent evidence has suggested that suboptimal levels of vitamin D have an adverse effect on the cardiovascular system and are associated with several pathways that lead to adverse cardiac remodeling, inflammation, and worsening chronic HF.^[1–3]^

Previous studies have demonstrated that vitamin D deficiency is often prevalent and associated with adverse clinical outcomes in patients with chronic HF.^[4,5]^ In recent years, several randomized controlled trials (RCTs) have evaluated the effects of vitamin D supplementation in patients with chronic HF.^[6–16]^ However, the results have been inconsistent and remain controversial.

The aim of the vascular and ventricular function of vitamin D in patients with chronic HF trial (VIVID-HF) was to examine whether vitamin D supplementation improves endothelial function and protects left ventricular function in patients with chronic and stable HF.

## 2. Methods

### 2.1. Study population

The VIVID-HF trial was an investigator-initiated, prospective, randomized placebo-controlled trial of vitamin D supplementation in vitamin D-deficient patients with chronic HF on optimal medical therapy. Patients were eligible if they had stable (>3 months) New York Heart Association (NYHA) class II or III symptoms and elevated biomarkers (NT-proBNP ≥ 400 pg/mL or BNP *>* 200 pg/mL) on maximally tolerated medical therapy (>3 months) and a 25(OH) vitamin D level of <75 nmol/L (<30 ng/mL). The definition of each etiology of heart failure was defined as follows: systolic HF was defined as an ejection fraction (EF) of ≤40%, diastolic HF as an EF of ≥50%, and nonischemic systolic HF as an EF of ≤40% with no significant obstructive coronary disease.

Patients were ineligible if they were taking or had taken calcium or other vitamin supplements in the last 3 months. Other exclusion criteria included a life expectancy of less than 1 year, significant valvular heart disease requiring surgery, myocardial infarction, unstable angina, or revascularization within 60 days; significant arrhythmia (ventricular tachycardia/fibrillation, complete atrioventricular block), primary pulmonary hypertension, other causes of clinically important dyspnea such as anemia or thyrotoxicosis, existing indications for vitamin D supplementation (e.g., previous osteoporotic fracture or symptoms of osteomalacia), a history of primary hyperparathyroidism, sarcoidosis, tuberculosis or lymphoma, a vitamin D concentration at the time of screening >75 nmol/L (30 ng/mL) or significant renal dysfunction (estimated glomerular filtration rate [eGFR] <30 mL/min), and, if female, being pregnant or breastfeeding. The patients provided written informed consent for participation in the trial. The study protocol was approved by the Gangneung Asan Hospital ethical committee of Ulsan University (reference GNAH IRB No. 2016-11-0007). The study protocol was performed in accordance with the principles of the Declaration of Helsinki.

**Figure 1.**
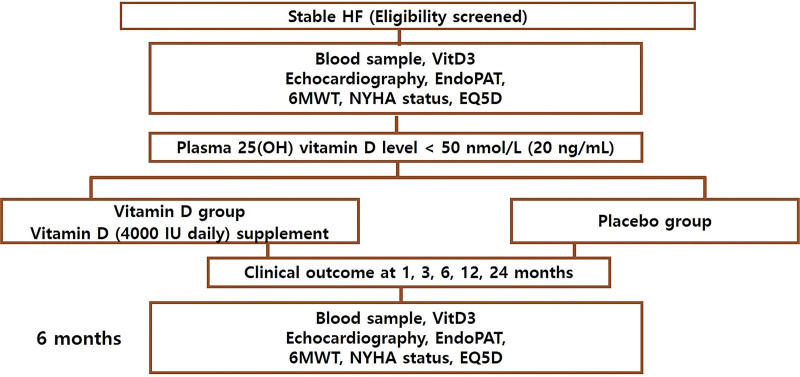
Study flow chart. Abbreviations: HF = heart failure; vitD3 = vitamin D3; PAT = pulse amplitude tonometry; 6MWT = 6-min walk test; NYHA = New York Heart Association.

Finally, eligible subjects were randomly assigned at a 1:1 ratio to each of the two groups (vitamin D or placebo group) for 6 months. Web-based computerized randomization was conducted at each participating site. The vitamin D group was asked to take a total of 100 μg 25(OH) vitamin D3 (4000 IU daily), and the placebo group was administered 1 tablet of placebo of vitamin D3, which was formulated for this study. The supplement and dose were chosen based on previous studies of vitamin D supplementation.^[16]^ As shown in Figure 1, each patient underwent pulse amplitude tonometry (PAT), the 6-min walking test (6 MWT), echocardiography, and blood sampling for serum calcium, serum creatinine, vitamin D, and parathyroid hormone (PTH) levels at baseline. Subsequent visits took place 6 months later, and blood tests were repeated at each visit.

### 2.2. Outcome variables

The prespecified primary endpoint in VIVID-HF was the difference in endothelial function assessed by PAT (baseline to 6 months) between the 2 groups. Key prespecified secondary endpoints included the difference in change in the 6 MWT distance (baseline to 6 months) between the 2 groups, symptoms (as assessed by the EQ5D questionnaire and NYHA classification), cardiac structure and function (as assessed by echocardiography), safety endpoints of serum calcium concentration, and vitamin D levels. Hypervitaminosis D was defined as >200 nmol/L (80 ng/mL) and hypercalcemia as >2.6 nmol/L (10.4 mg/dL).

### 2.3. Endothelial function measurement using PAT

PAT was assessed by the same examiner, who was blinded to the subjects’ clinical backgrounds. The digital pulse amplitude was measured with a PAT device placed on the tip of each index finger (Endo-PAT2000; Itamar Medical, Caesarea, Israel). The PAT device comprises a pneumatic plethysmograph that applies uniform pressure to the surface of the distal finger, allowing the measurement of pulse volume changes in the finger. Measurements were obtained continuously for 5 minutes at baseline, cuff occlusion, and postcuff deflation. The data were automatically derived using Endo-PAT version 3.2.4 software (Itamar Medical Ltd.). The digital PAT ratio was calculated as the ratio of post-cuff deflation pulse volume amplitude (PVA) (at 30 seconds intervals) to the baseline PVA in the arm undergoing cuff occlusion (occluded) and then normalized to the contralateral (control) arm. The digital PAT ratio in the 90–120 seconds postcuff deflation intervals (PVA occluded, 90–120 seconds/PVA occluded, baseline)/ (PVA control, 90–120 seconds/PVA control, baseline) is presented.^[17]^

### 2.4. Serum biochemistry

Using tandem mass spectrometry, serum 25(OH)D2 and 25(OH)D3 levels were analyzed. Samples were prepared using a protein precipitation reagent containing deuterated 25(OH)D3. The supernatant was analyzed using an API5000 LC-MS/MS system (AB SCIEX, Warrington, UK) in the APCI mode. The inter-assay CV was <10% at all concentrations ranging from 12 to 159 nmol/L (4.8–63.7. 25(OH)D2 and vitamin D3 concentrations were summed and reported as the total 25(OH)D concentration. Vitamin D deficiency and insufficiency were defined as <50 nmol/L (20 ng/mL) and <75 nmol/L (30 ng/mL), respectively.^[18,19]^ We also measured serum calcium, creatinine, and parathyroid hormone levels (Siemens Advia and Centaur, Siemens Healthcare Diagnostics, Camberley, UK). To confirm the effective conversion of the supplement, we also measured 1,25(OH)vitamin D3 using a radio-immuno-assay (IDS, Boldon, UK) at baseline and at 6 months.

### 2.5. Echocardiography

Echocardiography was performed for all patients at baseline and 6 months later. M-mode tracing obtained just below the mitral valve leaflets was acquired in the parasternal short-axis view. We measured LV end-diastolic (LVED) and end-systolic (LVES) dimensions, such as interventricular septal wall thickness, posterior wall thickness, and LVED and LVES diameters.^[20]^ LVED and LVES volumes were acquired from apical 2- and 4-chamber views using the biplane modified Simpson rule, while the LV EF was calculated according to American Society of Echocardiography (ASE) recommendations.^[20]^ Left atrial (LA) volume indices were also measured using the biplane Simpson method. LV mass was calculated using the Devereux formula and indexed to the body surface area. LV mass index and E/E’, calculated using the Mosteller formula, were assessed according to recordings based on the recommendations of the ASE/EACVI.^[20]^ In addition, speckle tracking analysis using the commercially available automated function imaging technique was applied to assess the global longitudinal strain (GLS) from the apical long-axis slices (long-axis and 2- and 4-chamber views) according to recordings based on the recommendations of the ASE/EACVI. Echocardiograms at both time points were analyzed offline at the end of the study by 2 senior echocardiographers blinded to the patient treatment group.

### 2.6. Statistical analysis

The value of EndoPAT in HF was 1.3 ± 0.2 in a preclinical study. A sample size of 92 patients (46 patients in each treatment group) was required to provide a statistical power of 80% and a probability of a type I error of 0.05, using a 2-sided test. A minimum of 110 patients should be registered, assuming a 20% dropout rate.

Continuous variables were presented as the mean ± standard deviation if normally distributed or as a median (25^th^–75th percentile) if not normally distributed. When appropriate, categorical variables, presented as frequencies and percentages, were compared using the chi-square test or Fisher exact test. The analysis of primacy for the main efficacy endpoints was based on analysis of covariance linear models relating differences in the final walk distance and imaging variables by treatment allocation after adjusting for baseline values and were reported with 95% confidence intervals.^[21]^ We performed a repeated-measures analysis of variance to determine the differences between groups and over time. All analyses were conducted using Stata software (StataCorp. 2015. *Stata Statistical Software: Release 14*. College Station, TX, StataCorp LP). All significance tests were 2-sided and considered significant at the 5% level.

## 3. Results

### 3.1. Study population and clinical follow-up

**Figure 2.**
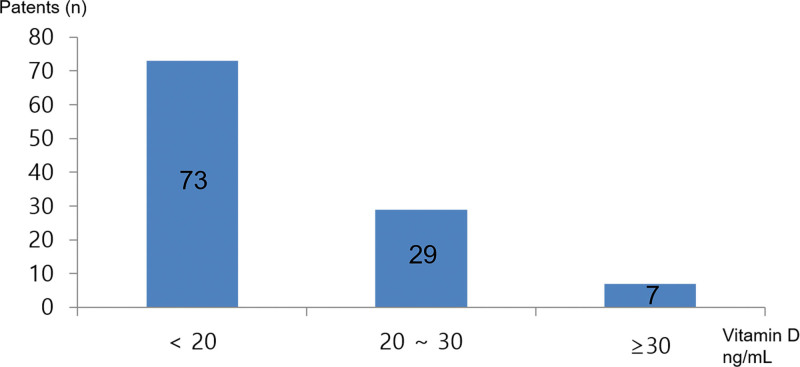
The prevalence of vitamin D deficiency during screening in our study. In total, 109 stable HF patients were screened from September 2016 and August 2017, and 73 (67%) patients showed vitamin D deficiency.

A total of 109 stable HF patients were screened from September 2016 to August 2017, and 73 (67%) of these patients showed vitamin D deficiency (Fig. 2). The etiology of HF (Table 1) was idiopathic dilated cardiomyopathy, ischemic systolic HF, nonischemic systolic HF, or diastolic HF. Seventy-three patients were enrolled in our study and were randomized into treatment (39 patients) or placebo (34 patients) groups. All patients completed the study at the 6-month follow-up. The baseline characteristics of the 2 groups divided by treatment allocation are shown in Tables 1 and 2. There were no significant clinical differences at baseline between patients who completed the study and those who dropped out. The 2 groups of participants were balanced for baseline clinical, laboratory, and hemodynamic variables (Tables 1 and 2). Vitamin D3 supplementation was well tolerated, and therefore, there was no follow-up loss or major side effects in either group.

**Table 1:** Baseline clinical characteristics.

	Overall (n = 73)	VitD (n = 39)	Placebo (n = 34)	*P* value
Clinical variables				
Age, yr	66.0 ± 9.6	66.4 ± 7.2	66.5 ± 11.8	0.72
Male sex, n (%)	31 (42%)	18 (46%)	13 (38%)	0.64
Body mass index (kg/m^2^)	23.5 ± 3.3	23.6 ± 3.1	23.9 ± 3.4	0.71
Diabetes mellitus	25 (34%)	14 (36%)	11 (32%)	0.81
Hypertension	51 (67%)	18 (46%)	13 (38%)	0.64
CAD	27 (16%)	16 (41%)	11 (32%)	0.48
CVA	16 (23%)	7 (19%)	9 (28%)	0.40
AF	29 (40%)	16 (41%)	13 (38%)	0.99
CKD	29 (42%)	18 (46%)	11 (32%)	0.33
Etiology of HF				0.12
Idiopathic DCMP	24 (33%)	9 (23%)	15 (44%)	
Ischemic systolic HF	21 (29%)	14 (36%)	7 (21%)	
Nonischemic systolic HF	16 (22%)	11 (28%)	5 (15%)	
Diastolic HF	12 (16%)	5 (13%)	7 (21%)	
Medications				
Beta blocker	62 (85%)	34 (87%)	28 (82%)	0.74
RAS inhibitor	63 (86%)	33 (85%)	30 (88%)	0.74
Digoxin	7 (10%)	4 (10%)	3 (9%)	0.99
Furosemide (mg)	38.7 ± 29.3	38.9 ± 23.4	38.2 ± 35.3	0.92
Spironolactone	36 (49%)	22 (56%)	14 (41%)	0.24

Data, if appropriate, are presented as the mean ± SD. Significant values are shown in bold.

Abbreviations: AF = atrial fibrillation, CAD = coronary artery disease, CKD = chronic kidney disease, CVA = cerebrovascular accident, DCMP = dilated cardiomyopathy, HF = heart failure, RAS = renin-angiotensin system, VitD = vitamin D.

**Table 2:** Baseline laboratory and hemodynamic characteristics.

	Overall (n = 73)	VitD (n = 39)	Placebo (n = 34)	*P* value
Laboratory parameters				
Hb (g/dL)	11.9 ± 2.1	11.9 ± 2.2	12.1 ± 1.9	0.80
BUN (mg/dL	17.5 ± 11.6	18.1 ± 12.4	16.8 ± 10.8	0.62
Creatinine (mg/dL)	0.9 ± 0.5	1.0 ± 0.6	0.9 ± 0.4	0.42
Calcium (mg/dL)	8.7 ± 0.5	8.7 ± 0.6	8.9 ± 0.4	0.07
Phosphate (mg/dL)	3.6 ± 0.7	3.7 ± 0.7	3.4 ± 0.6	0.17
Total cholesterol (mg/dL)	173.9 ± 40.9	176.7 ± 47.0	170.7 ± 32.9	0.54
Triglyceride (mg/dL)	130.4 ± 76.8	153.6 ± 94.3	112.2 ± 40.9	0.02
LDLcholesterol (mg/dL)	101.3 ± 34.4	107.3 ± 40.8	93.9 ± 23.0	0.12
HDL cholesterol (mg/dL)	46.2 ± 12.5	43.8 ± 12.9	49.1 ± 11.5	0.08
HbA1c (%)	6.5 ± 0.9	6.5 ± 0.9	6.5 ± 0.9	0.33
NT-proBNP (pg/mL)	938.6 ± 622.5	989.5 ± 671.1	880.3 ± 566.0	0.46
Vitamin D (nmol/L)	12.6 ± 3.2	11.8 ± 3.2	13.3 ± 3.0	**0.04**
Hemodynamic parameters				
SBP (mm Hg)	119.5 ± 19.0	122.2 ± 15.6	116.4 ± 22.1	0.20
DBP (mm Hg)	71.8 ± 13.5	73.6 ± 13.9	69.9 ± 12.8	0.25
HR (beat/min)	74.9 ± 15.8	76.1 ± 16.3	73.6 ± 15.5	0.53

Data, if appropriate, are presented as the mean ± SD. Value shown in bold represents significance.

Abbreviations: BNP = B-type natriuretic peptide, BUN = blood urea nitrogen, DBP = diastolic blood pressure, LDL = low-density lipoprotein, Hb = hemoglobin, HDL = high-density lipoprotein, HbA1c = hemoglobin A1c, HR = heart rate, SBP = systolic blood pressure.

**Figure 3.**
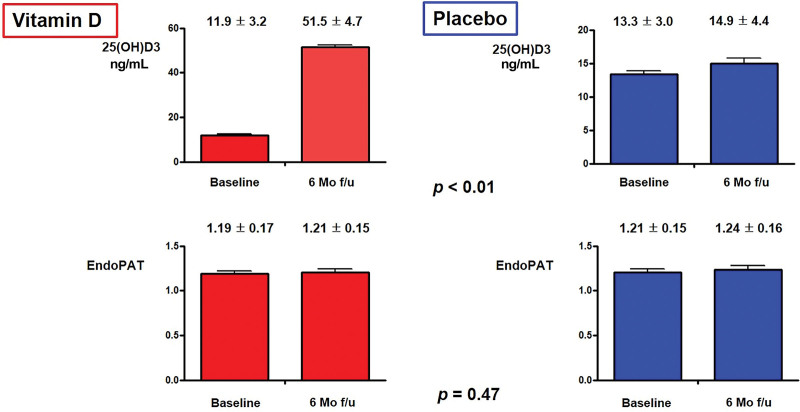
Changes in vitamin D and endothelial function at baseline and at the 6-month follow-up. Abbreviations: PAT = pulse amplitude tonometry.

Vitamin D3 supplementation led to sustained normal serum 25(OH)D concentrations by 6 months postrandomization, indicating excellent adherence to treatment and maintenance of a safe concentration (Fig. 3).

### 3.2. Clinical outcomes after vitamin D supplementation

**Figure 4.**
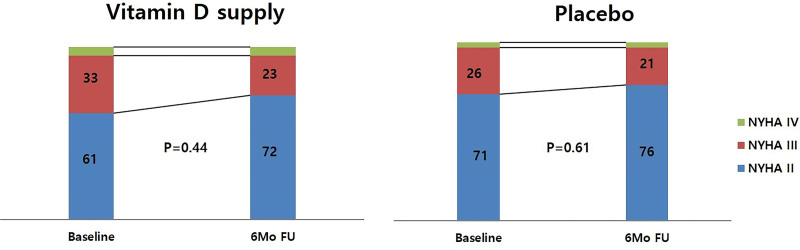
Change in NYHA class at baseline and at the 6-month follow-up. Abbreviations: NYHA = New York Heart Association. The numbers in the graph show the proportions (%) of each group.

Table 3 summarizes the baseline blood pressure and symptomatic parameters and the differences in responses to the 2 treatment allocations. As shown in Figure 3, 6-month treatment with 4000 IU cholecalciferol did not improve endothelial function assessed by EndoPAT in patients with chronic HF (baseline: 1.19 ± 0.17 vs 6 months: 1.21 ± 0.15, *P* = .47) even though vitamin D supplementation normalized vitamin D levels (baseline: 11.9 ± 3.2 vs 6 months: 51.5 ± 4.7, *P* < .01). However, at 6 months, as shown in Table 3, patients in the vitamin D group showed significant improvement in the 6 MWT distance compared with the placebo group (vitamin D group, from 152.9 ± 31.9 to 194.4 ± 40.5 m, *P* < .01 vs the placebo group, from 145.0 ± 31.6 to 209.9 ± 51.2 m, *P* = .11). The vitamin D group also exhibited a significant improvement in quality of life as assessed by the EQ-5D questionnaire (vitamin D group, from 8.4 ± 1.7 to 7.7 ± 2.0, *P* < .01 vs placebo group, from 8.0 ± 1.4 to 7.8 ± 2.3, *P* = .44) and Borg scale > 5 (vitamin D group, 19 (48%) to 4 (17%), *P* = .03 vs placebo group, from 14 [41%] to 4 [19%], *P* = .095). The proportion of NYHA class numerically improved after 6 months, particularly in the vitamin D group compared with the placebo group (Fig. 4).

**Table 3:** Changes in the clinical outcomes between the baseline and 6 months vitamin D administration.

	Vitamin D			Placebo	
	Baseline	6 Mo	Baseline	6 Mo	*P* value
S BP (mm Hg)	**122.2 ± 15.6**	**110.9 ± 15.8** *	116.4 ± 22.1	112.1 ± 13.8	0.30
D BP (mm Hg)	**73.6 ± 13.9**	**63.4 ± 18.8** *	69.9 ± 12.8	68.3 ± 15.3	0.81
HR (beat/min)	76.1 ± 16.3	72.2 ± 12.3	73.6 ± 15.5	73.1 ± 14.7	0.74
6 MWD (m)	**152.9 ± 31.9**	**194.4 ± 40.5** *	145.0 ± 31.6	209.9 ± 51.2	0.60
Borg scale ≥ 5	**19 (48%**)	**4 (17%**)*	14 (41%)	4 (19%)	0.25
NYHA class					0.57
II	24 (61%)	28 (72%)	24 (71%)	26 (76%)	
III	13 (33%)	9 (23%)	9 (26%)	7 (21%)	
IV	2 (5%)	2 (5%)	1 (3%)	1 (3%)	
EQ5D	**8.4 ± 1.7**	**7.7 ± 2.0** *	8.0 ± 1.4	7.8 ± 2.3	0.85
Mobility ≥ 2	21 (56%)	15 (40%)	13 (56%)	9 (39%)	0.99
Self-care ≥ 2	29 (78%)	21 (60%)	18 (78%)	14 (61%)	0.68
Usual activities ≥ 2	33 (95%)	25 (68%)	20 (87%)	15 (65%)	0.32
Pain ≥ 2	18 (49%)	13 (35%)	10 (43%)	8 (35%)	0.81
Anxiety ≥ 2	11 (30%)	14 (38%)	7 (30%)	12 (52%)	0.52

Data, if appropriate, are presented as the mean ± SD. Significant values are shown in bold.

* , *P < .05*, between the baseline and after 6 months of follow up within each group; *P* value shows the comparison of changes between the Vitamin D and placebo groups during the 6 month follow-up period.

Abbreviations: DBP = diastolic blood pressure, HR = heart rate, 6WMD = 6-min walking distance, NYHA = New York Heart Association, SBP = systolic blood pressure.

### 3.3. Impact of vitamin D on heart remodeling

At 6 months, there was no significant change in LV chamber size in the vitamin D group (left ventricular end-diastolic dimension: vitamin D group, 54.9 ± 8.5 to 50.2 ± 19.2 vs the placebo group, 57.6 ± 7.6 to 54.2 ± 13.4 mm, *P* = .11; left ventricular end-systolic dimension: vitamin D group, 43.3 ± 9.1 to 42.9 ± 8.6 mm vs the placebo group, 45.8 ± 8.1 to 44.4 ± 9.2, *P* = .16) as shown in Table 4. LVEF and LV GLS did not change either (LVEF: vitamin D group, 41.6 ± 10.0 to 41.8 ± 10.7 vs placebo group, 39.7 ± 10.5 to 40.6 ± 10.9%, *P* = .54; LV GLS: vitamin D group, -10.3 ± 2.5 to -10.6 ± 3.0 vs placebo group, -10.1 ± 2.9 to -10.4 ± 3.1%, *P* = .69). However, there was a significant decrease in the LA diameter in the vitamin D group (vitamin D group, 41.1 ± 6.5 to 38.7 ± 6.6, *P* = .04 vs placebo group, 40.8 ± 8.6 to 39.4 ± 7.1 mm, *P* = .94). From a hemodynamic perspective, there was a significant decrease in systolic and diastolic blood pressure compared with patients in the placebo group (systolic blood pressure: vitamin D group, 122.2 ± 15.6 to 110.9 ± 15.8, *P* < .01 vs the placebo group, 116.4 ± 22.1 to 112.1 ± 13.8 mm Hg, *P* = .43; diastolic blood pressure: vitamin D group, 73.6 ± 13.9 to 63.4 ± 18.8, *P* < .01 vs the placebo group, 69.9 ± 12.8 to 68.3 ± 15.3 mm Hg, *P* = .66; Table 3).

**Table 4:** Changes in the echocardiographic parameters between baseline and 6 months administration of vitamin D.

	Vitamin D			Placebo	
	Baseline	6 Mo	Baseline	6 Mo	*P* value
LV end-diastolic dimension (mm)	54.9 ± 8.5	50.2 ± 19.2	57.6 ± 7.6	54.2 ± 13.4	0.11
LV end-systolic dimension (mm)	43.3 ± 9.1	42.9 ± 8.6	45.8 ± 8.1	44.4 ± 9.2	0.16
LV end-diastolic volume (ml)	111.7 ± 42.1	117.9 ± 55.4	111.5 ± 46.5	119.7 ± 60.4	0.84
LV end-systolic volume (ml)	66.0 ± 31.9	71.4 ± 42.2	68.2 ± 34.6	70.9 ± 46.2	0.86
LV mass index (g/m^2^)	123.3 ± 37.4	139.5 ± 43.9	131.6 ± 37.2	137.7 ± 44.8	0.85
Ejection fraction (%)	41.6 ± 10.0	41.8 ± 10.7	39.7 ± 10.5	40.6 ± 109	0.54
Global longitudinal strain (%)	–10.3 ± 2.5	–10.6 ± 3.0	–10.1 ± 2.9	–10.4 ± 3.1	0.69
E/e’	19.6 ± 9.9	18.5 ± 8.1	18.1 ± 8.1	17.7 ± 7.4	0.26
LA diameter (mm)	**41.1 ± 6.5**	**38.7 ± 6.6** *	40.8 ± 8.6	39.4 ± 7.1	0.94
LA volume index (ml/m^2^)	38.9 ± 9.1	36.3 ± 12.7	38.9 ± 8.9	34.4 ± 17.5	0.91

Data, if appropriate, are presented as the mean ± SD. Significant values are shown in bold.

* *P* < .05, between the baseline and 6 month follow up within each group; *P* value shows the comparison of changes between Vitamin D group and placebo group during the 6 month follow-up period.

Abbreviations: LV = left ventricular, E/e’ = E wave to e’ ratio, LA = left atrial.

## 4. Discussion

Our study sought to examine the effect of high-dose vitamin D3 supplementation in patients with various types of chronic HF (including ischemic, nonischemic, idiopathic, systolic, and diastolic HF) who underwent guideline-directed optimal medical therapy. Although vitamin D supplementation did not affect the primary endpoint of endothelial function assessed by EndoPAT, there were statistically significant, prognostically, and clinically relevant improvements in the secondary outcomes of 6 MWT distance and quality of life when 4000 IU vitamin D3 was administered daily for 6 months. High-dose vitamin D supplementation was safe, well tolerated, and not associated with adverse biochemical effects when administered with HF guideline-directed medical therapies. In addition, vitamin D supplementation was not associated with remodeling of the LV, but there was a tendency toward improvement in the LA diameter, suggesting that vitamin D is associated with beneficial LA reverse remodeling.

Vitamin D deficiency occurs in approximately 90% of patients with chronic HF,^[22]^ although it is less frequent in our study population (67%). Low vitamin D levels activate the renin-angiotensin-aldosterone system, induce an inflammatory response, and cause endothelial dysfunction.^[1–3]^ Therefore, chronic HF is closely associated with vitamin D levels, and several clinical trials have demonstrated that vitamin D deficiency is prevalent and associated with poor prognosis in patients with chronic HF.^[4,5]^ The VINDICATE study with chronic HF secondary to LV systolic dysfunction showed that high-dose vitamin D supplementation might mitigate adverse LV remodeling and improve LV remodeling.^[9]^ A pathophysiological hallmark of chronic HF secondary to LV systolic dysfunction is a progressive increase in LV cavity dimensions and impaired contractility, a process known as LV remodeling.^[23]^ The degree of favorable remodeling-induced HF guideline-directed optimal medical therapy is related to long-term outcomes.^[18]^ Therefore, it is plausible that the improvements in cardiac function demonstrated in VINDICATE have the potential to improve outcomes.

A meta-analysis of observational data indicated a statistically positive association between plasma calcium levels and cardiovascular disease.^[19]^ More importantly, the Atherosclerosis Risk in Communities (ARIC) study reported that high plasma calcium was independently associated with a greater risk of incident HF.^[24]^ In that study, HF incidence was lowest at calcium levels of 2.25 mmol/L and increased progressively up to 2.75 mmol/L.^[19,24]^ However, a recent meta-analysis of RCTs demonstrated that vitamin D supplementation in patients with chronic HF exerted no beneficial effects on mortality and left ventricular function but could improve quality of life.^[25]^ In contrast to the meta-analysis, 2 smaller RCTs reported a significant increase in LVEF with vitamin D doses equivalent to 4000 IU daily. However, these RCTs did not include patients who dropped out or violated the study protocol in their data analysis. Moreover, they did not assess clinical events.^[9,10]^ In the EVITA study, which enrolled patients with advanced HF, a daily vitamin D dose of 4000 IU did not reduce mortality but was associated with a greater need for mechanical circulatory support implants. In summary, vitamin D supplementation did not improve LV function in previous studies on HF.^[6]^

In our study, calcium levels were stably maintained during follow-up. Despite the lack of beneficial effects on endothelial function and cardiac remodeling, especially LV structure, there was some impact on LA size. This suggests that vitamin D supplementation has a positive effect on atrial remodeling. LA structural remodeling is a complex phenotypic expression that results from changes in LA size, shape,^[26]^ and architecture and alterations in the cardiomyocyte, fibroblast, and noncollagen infiltrative compartments of the atrium.^[27]^ LA enlargement, which is simple to measure, is the default clinical hallmark of structural remodeling that occurs most often in response to LA pressure and volume overload. In the absence of atrial fibrillation (AF), mitral valvular disease, and high cardiac output states, it is an excellent biomarker for the presence and severity of LV diastolic dysfunction.^[28]^ Moreover, LA enlargement is associated with a poor prognosis in various cardiovascular diseases.^[23,26–29]^ In the HF setting, the LA dimension was higher in the AF than in the sinus rhythm and predicted AF. Furthermore, LA dilation was associated with the severity of HF in HF with preserved EF, HF mid-range EF, and HF reduced EF and predicted the progression to HF reduced EF.^[29,30]^

Although the patients in the VINDICATE study had more advanced HF and remodeled LV (mean EF, 25.6 %; LVEDD, 58 mm; LVESD, 48 mm), our study population had higher EF (mean EF = 40.7%) and less remodeled LV (LVEDD/LVESD = 56/44 mm). In addition, from an etiologic point of view, one of the HF etiologies in our study population was diastolic heart failure (EF normal but diastolic dysfunction and HF symptoms). The VINDICATE study focused on LV remodeling, but our study duration was too short for LV remodeling. Therefore, due to the shorter study duration and less remodeled LV patient population, there was a lack of beneficial effects on LV structure. However, LA structural remodeling was prominent, which led to symptom improvement and increased the 6 minutes walk distance.

Data reported previously show that rehospitalizations for HF after release are estimated to be 6%, intrahospital mortality 6%, and annual mortality appears to exceed 24%. Access to hospitals and the consumption of resources required for the management of patients with chronic HF are still high.^[31,32]^ Evidence suggests that vitamin D supplementation may have a positive role in the management of chronic HF. Vitamin D supplementation may show good results if combined with optimal medical therapy in terms of improved cardiac performance and greater physical fitness in patients with chronic HF and vitamin D deficiency. Studies that test the real effects and benefits of vitamin D supplementation on outcomes in patients with chronic HF are needed. Therefore, this research shows that new low-cost approaches to therapy may be possible in the future. These approaches could reduce hospitalizations and provide additional advantages with regard to quality of life and the rational use of available resources in hospitals.^[33]^

This study has several limitations. First, this study included only a small number of patients and had a relatively short follow-up period. Second, the etiology of HF was heterogeneous. Third, we did not perform central BP monitoring that would consider seasonal variation in vitamin D. Fourth, we did not assay other biomarkers related to vitamin D metabolism or sex hormones. Our study was based on results from a multicenter, randomized, placebo-controlled study in 39 patients using the same dose for 6 months. However, our study only showed a favorable effect of vitamin D on LA remodeling. We did not examine the effect of vitamin D supplementation in patients with chronic HF and preserved EF, a group of patients who may warrant such investigations. Despite the study limitations, vitamin D supplementation might be an adjunctive therapy for use with guideline-directed optimal HF therapy, and future studies are needed to confirm its effects in HF therapy.

## 5. Conclusions

In conclusion, a daily vitamin D dose of 4000 IU for chronic HF is safe. This dose did not improve endothelial function but did improve the 6-min walk distance, symptoms, and LA diameter at 6 months. Therefore, high-dose vitamin D administration may be a safe and adjunctive strategy for treating chronic HF.

## Acknowledgment

We would like to thank Editage (www.editage.co.kr) for English language editing.

## Author contributions

Clinical studies: Jong Shin Woo

Data acquisition: Jong Shin Woo

Data analysis: Jong Shin Woo

Definition of intellectual content: Sang Jin Ha

Experimental studies: Sang Jin Ha

Guarantor of integrity of the entire study: Sang Jin Ha

Literature research: Sang Jin Ha

Manuscript editing: Yeongmin Woo

Manuscript preparation: Sang Jin Ha

Manuscript review: Jeong Yoon Jang

Statistical analysis: Jong Shin Woo

Study concepts: Sang Jin Ha

Study design: Jong Shin Woo
